# Metabolic reprogramming: the emerging concept and associated therapeutic strategies

**DOI:** 10.1186/s13046-015-0221-y

**Published:** 2015-10-06

**Authors:** Go J. Yoshida

**Affiliations:** Research Fellow of Japan Society for the Promotion of Science, Tokyo, Japan; Department of Pathological Cell Biology, Medical Research Institute, Tokyo Medical and Dental University, 1-5-45 Yushima, Bunkyo-ku, Tokyo 113-8510 Japan

**Keywords:** Intra-tumoral heterogeneity, Cancer stem-like cells, Minimal residual disease, Reverse Warburg effect, Cancer-associated fibroblasts, Metabolic symbiosis, Glutaminolysis, AMPK-mTOR signal, Drug-repositioning, Metformin

## Abstract

Tumor tissue is composed of cancer cells and surrounding stromal cells with diverse genetic/epigenetic backgrounds, a situation known as intra-tumoral heterogeneity. Cancer cells are surrounded by a totally different microenvironment than that of normal cells; consequently, tumor cells must exhibit rapidly adaptive responses to hypoxia and hypo-nutrient conditions. This phenomenon of changes of tumor cellular bioenergetics, called “metabolic reprogramming”, has been recognized as one of 10 hallmarks of cancer. Metabolic reprogramming is required for both malignant transformation and tumor development, including invasion and metastasis. Although the Warburg effect has been widely accepted as a common feature of metabolic reprogramming, accumulating evidence has revealed that tumor cells depend on mitochondrial metabolism as well as aerobic glycolysis. Remarkably, cancer-associated fibroblasts in tumor stroma tend to activate both glycolysis and autophagy in contrast to neighboring cancer cells, which leads to a reverse Warburg effect. Heterogeneity of monocarboxylate transporter expression reflects cellular metabolic heterogeneity with respect to the production and uptake of lactate. In tumor tissue, metabolic heterogeneity induces metabolic symbiosis, which is responsible for adaptation to drastic changes in the nutrient microenvironment resulting from chemotherapy. In addition, metabolic heterogeneity is responsible for the failure to induce the same therapeutic effect against cancer cells as a whole. In particular, cancer stem cells exhibit several biological features responsible for resistance to conventional anti-tumor therapies. Consequently, cancer stem cells tend to form minimal residual disease after chemotherapy and exhibit metastatic potential with additional metabolic reprogramming. This type of altered metabolic reprogramming leads to adaptive/acquired resistance to anti-tumor therapy. Collectively, complex and dynamic metabolic reprogramming should be regarded as a reflection of the “robustness” of tumor cells against unfavorable conditions. This review focuses on the concept of metabolic reprogramming in heterogeneous tumor tissue, and further emphasizes the importance of developing novel therapeutic strategies based on drug repositioning.

## Introduction

Tumor tissue consists of a heterogeneous cellular population. Stromal cells such as neurons, vascular endothelial cells, fibroblasts, and macrophages in cancer tissue drive chemotherapy resistance [1] as well as tumor survival and progression [2, 3]. Even in pure populations of tumor cells, heterogeneity is present as a result of genetic mutation and epigenetic modulations. This cellular heterogeneity can be explained by a hierarchical model, in which cancer stem-like cells (CSCs) can provide transient amplifying cells and differentiated non-CSCs involved in establishing the tumor tissue [4, 5]. CSCs possess several biological features of “stemness”, a combination of phenotypes including plasticity in the transition between quiescent (G_0_ phase) and proliferative states [6] and resistance to redox stress and chemotherapeutic agents [7, 8]. Importantly, accumulating evidence suggests that metabolic reprogramming is crucial in order for CSCs to maintain unlimited self-renewal potential and hyper-adaptation to drastic changes in the tumor microenvironment [9–11].

**Fig. 1:**
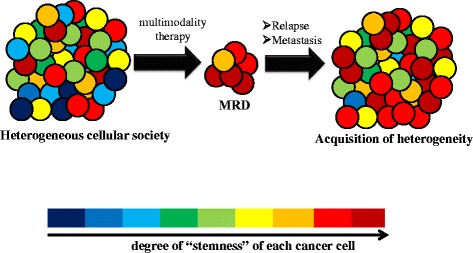
Cancer stem cells and MRD formation. Heterogeneous tumor tissue with combined-modality therapy leads to the formation of MRD, which is clinically undetectable. Transiently reduced heterogeneity is observed in MRD, which is enriched in CSCs. Relapse or metastasis results in re-acquisition of a heterogeneous population that is more potentially aggressive in terms of its degree of “stemness”

Intra-tumoral heterogeneity due to the presence of CSCs is primarily responsible for our inability to induce the same therapeutic effect among cancer cells as a whole [12, 13]. CSCs are very likely to contribute to the formation of minimal residual disease (MRD) [1]. The term ‘MRD’ is most often used in the context of hematological malignant disorders [14], but the underlying concept is quite convenient in discussion of clinically undetectable resistant clones after conventional anti-tumor therapies [1]. Thus, MRD is expected to contribute prominently to latent relapse and distant metastasis (Fig. 1).

Aberrant proliferation of cancer cells is supported by enhanced adaptation to nutrient microenvironment mediated by alterations in energy metabolism. Consequently, metabolic reprogramming is believed to be one of the hallmarks of tumor cells in parallel with genomic instability, tumor-provoking chronic inflammation, escape from the immune system, etc. [5]. Although aerobic glycolysis, termed the Warburg effect, is a characteristic metabolic feature of cancer cells [15, 16], recent investigations revealed that other metabolic features, in particular, the reverse Warburg effect [17, 18], metabolic symbiosis [19, 20], and addiction to glutamine metabolism [21, 22], create challenges for anti-cancer treatment due to adaptive or acquired chemoresistance. This review article focuses on the relationship between metabolic reprogramming and tumor heterogeneity, as well as on the development of promising therapeutic strategies by drug repositioning targeting metabolic reprogramming.

### Conventional Warburg effect and emerging concepts

In 1924, Otto Warburg discovered that tumor cells tend to produce large amounts of lactate from glucose, regardless of the available oxygen level [15, 16]. This situation is similar to anaerobic glycolysis, implying that oxidative phosphorylation (OXPHOS) is replaced by glycolysis in normal differentiated cells under hypoxia [23, 24]. However, cancer cells appear to engage in glycolytic metabolism before they are exposed to hypoxic conditions [15, 16]. OXPHOS in mitochondria generates as many as 36 mol ATP from 1 mol glucose, whereas the conversion of glucose to pyruvate or lactate produces only 2 or 4 mol ATP, respectively [25, 26]. It remains unclear why cancer cells largely depend on this “inefficient” metabolic pathway, even when enough oxygen is available [27, 28]. In striking contrast to normal cells, cancer cells preferentially uptake and convert glucose into lactate even in the presence of sufficient oxygen [29]. This seemingly “inefficient” metabolic characteristic relies largely on aberrant upregulation of GLUT1, a glucose transporters abundantly expressed in cancer cells [30, 31], although one contradictory study reported that GLUT1 is not necessarily involved in the Warburg effect depending on the degree of tumor invasiveness [32]. Inefficient ATP synthesis becomes an obstacle for cancer cells only when their energy resources are scarce. However, this is not the case in proliferating cancer cells with aberrant angiogenesis [29]. Tumor cells finely regulate ATP synthesis by regulating substrate uptake, as well as enzymes related to glycolysis, which enables them adapt to the nutrient microenvironment [33]. Moreover, the regulation of adenosine monophosphate-activated protein kinase (AMPK) signal transduction, a sensor of energy status, is intimately connected to the Warburg effect, one form of metabolic reprogramming of cancer cells [34, 35]. Indeed, genetic ablation of AMPK activates mammalian target of rapamycin (mTOR) signal with ectopic expression of hypoxia-inducible factor-1 alpha (HIF-1 alpha), resulting in rapid cellular proliferation accompanied by activation of aerobic glycolysis [35]. This strongly suggests the importance of cancer metabolic reprogramming in maintaining the interaction between the oxygen-sensing transcription factor and the nutrient-sensing signal pathway.

### Metabolic reprogramming in response to chemotherapy

**Fig. 2:**
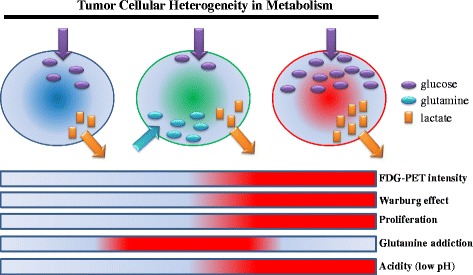
Tumor heterogeneity in metabolism. The degree of addiction to glucose or glutamate differs among various types of cancer cells. Tumor cells robustly importing glucose via the GLUT1 transporter are responsible for the high intensity of FDG-PET in the clinical settings. Cancer cells that express high levels of GLUT1 also induce a low-pH acidic tumor microenvironment, thereby increasing the invasive potential of tumors

**Fig. 3:**
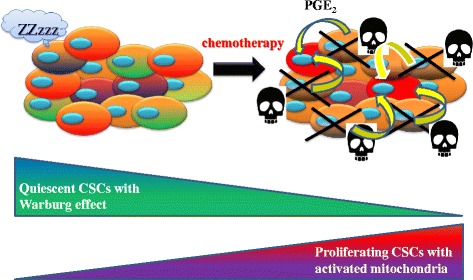
Iatrogenic activation of CSCs with altered metabolic reprogramming. Non-CSCs are susceptible to chemotherapy and undergo apoptosis. Released PGE_2_ awakens the dormant CSCs localized in the niche. Proliferating CSCs are likely to exhibit additional metabolic reprogramming, concomitant with up-regulation of OXPHOS-related molecules

Tumor heterogeneity in regard to mitochondrial metabolism, in seeming contradiction to the Warburg effect, is considered to induce the diversity in activated metabolic pathways [36] (Fig. 2). Notably, MRD in several kinds of cancers is enriched in CSCs, leading to intra-tumoral heterogeneity and poor prognosis [1, 9, 10, 37]. Non-CSCs of bladder cancer, for instance, release prostaglandin E_2_ (PGE_2_) when they undergo apoptosis during the course of chemotherapy. PGE_2_ promotes the awakening of dormant G_0_-phased CSCs into the proliferative state [9]. Given that PGE_2_-mediated metabolic activation in mitochondria has been demonstrated in non-malignant cells [38], it is possible that activated CSCs undergo altered metabolic reprogramming (Fig. 3). Similarly, the survivors after transient depletion of a driver oncogene (i.e., activated mutant *KRAS*^G12D^ in pancreatic cancer) tend to depend heavily on OXPHOS in mitochondria rather than aerobic glycolysis. Comprehensive analysis of metabolic pathways of survivors after chemotherapy revealed the prominent expression of genes that regulate mitochondrial function, autophagy and lysosome degradation activity, as well as a strong reliance on mitochondrial respiration and diminished dependence on the Warburg effect [10]. Autophagy is a metabolic-recycling pathway involving proteasome-independent degradation of cellular components (e.g., old and dysfunctional mitochondria), which is partially responsible for cancer chemoresistance [39].

Furthermore, malignant melanoma cells that survive and proliferate after treatment with mutant BRAF (V600E) inhibitor tend to exhibit relative dependence on mitochondrial metabolism [11]. Because BRAF suppresses oxidative phosphorylation (OXPHOS), MRD cells up-regulate proliferator-activated receptor-gamma coactivator-1 (PGC1-alpha). The BRAF (V600E)-MITF-PGC1-alpha axis promotes the biogenesis of mitochondria and causes BRAF-mutant melanoma cells to become addicted to mitochondrial metabolism [11]. Because histone H3 lysine 4 (H3K4)-demethylase JARID1B-highly expressing melanoma cells proliferate slowly and are highly dependent on mitochondrial metabolism [11, 40], chemotherapy-induced metabolic reprogramming in tumor tissue is likely to be responsible for the enrichment of CSCs in MRD.

### Metabolic interaction driven by tumor heterogeneity

**Fig. 4:**
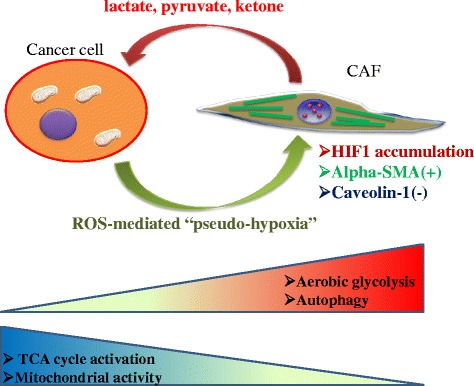
Interaction of caveolin 1-deficient CAFs with tumor cells. Cancer cells induce a pseudo-hypoxic microenvironment rich in ROS derived from metabolic reprogramming. By contrast, CAFs negative for caveolin 1 provide tumor cells with lactate, pyruvate, and ketone bodies. Notably, although cancer cells depend heavily on mitochondrial metabolism, CAFs exhibit the Warburg effect and activation of the autophagic pathway

Initially, the concept of Warburg effect was believed to be confined to cancer cells. More recently, the emerging concept of the “reverse Warburg effect”, however, has attracted considerable attention. Tumor cell-derived reactive oxygen species (ROS) decrease the expression of caveolin-1 in cancer-associated fibroblasts (CAFs). CAFs are the major component of tumor stroma, and as such they express alpha-smooth muscle actin (alpha-SMA) and are widely recognized to drive tumor progression and metastasis [41]. Loss of caveolin-1 in CAFs results in elevated ROS levels, which in turn stabilize  HIF-1 alpha [17, 42]. In brief, cancer cells create “pseudo-hypoxic” conditions for fibroblasts. Because the transcription factor HIF-1 alpha promotes glycolysis and provides tumor cells with lactate and glutamate, elevated production of ROS in cancer cells indirectly induces uptake of intermediate metabolites of the tricarboxylic acid (TCA) cycle in mitochondria. CAFs consume more glucose and secrete more lactate than normal fibroblasts. Furthermore, CAFs depend significantly on autophagy, and the activation of autophagy in tumor stroma leads to chemoresistance [18, 42] (Fig. 4).

As mentioned above, fibroblasts surrounding epithelial cancer cells undergo metabolic reprogramming resembling the phenotype associated with the Warburg effect. Metabolic symbiosis between epithelial cancer cells and CAFs requires that each cell express a different subtype of monocarboxylate transporter (MCT). Epithelial cancer cells express MCT1, which contributes to uptake of lactate provided by caveolin1-null CAFs expressing MCT4 [17, 43]. Tumor cells synthesize pyruvate from lactate, providing the TCA cycle with an intermediate metabolite. Notably, an extracellular space rich in lactate reflects acidic conditions, which in turn lead to the formation of pseudo-hypoxic conditions.

**Fig. 5:**
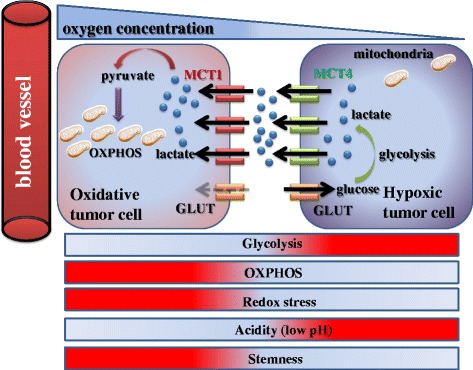
Metabolic symbiosis between oxidative/aerobic tumor cells and hypoxic/glycolytic cells. Tumor heterogeneity induces a lactate shuttle between hypoxic and oxidative cancer cells. While MCT4-positive hypoxic cells contribute to formation of an acidic microenvironment by aerobic glycolysis and secretion of lactate, MCT1-expressing oxidative cells utilize lactate as a substrate of the TCA cycle, and consequently exhibit stem-like characteristics. Notably, in contrast with MCT1-positive cancer cells, glucose uptake is robust in MCT4-expressing cells

It should be emphasized, however, that this reverse Warburg effect is not necessarily present in all tumor types. Tumors expressing high levels of MCT4 or mesenchymal phenotype do not tend to exhibit the reverse Warburg phenomenon. Instead, cancer cells exhibit hierarchical metabolic heterogeneity: MCT4-expressing tumor cells perform glycolysis and secrete lactate via MCT4, whereas MCT1-expressing cells import lactate via MCT1 and perform OXPHOS. In addition, the amount of glucose uptake is lower in MCT1-positive cancer cells than in MCT4-positive cells [19, 20] (Fig. 5). This metabolic heterogeneity is referred to as metabolic symbiosis, and this kind of lactate shuttle is also observed between neurons and astrocytes in the normal brain tissue [44]. It is notable that normal and cancerous tissues share finely regulated mechanisms of metabolic symbiosis.

### Cancer stem-like cells in metabolic symbiosis

Importantly, well-oxygenated/aerobic cancer cells expressing high levels of MCT1 efficiently produce metabolic intermediates, as well as ATP, by utilizing lactate derived from hypoxic/glycolytic cells expressing high levels of MCT4. Redox stress is a major hallmark of cancer tissues that drives robust metabolism in adjacent proliferating MCT1-positive cancer cells, which are rich in mitochondria, mediated by the paracrine transfer of mitochondrial fuels such as lactate, pyruvate, and ketone bodies [19, 20] (Figs. 4 and 5).

Most importantly, genotoxic stress due to chemotherapy or irradiation, which increase ROS levels, promotes a CSC-like phenotype [45–47]. Because CSCs exhibit a rapidly proliferating and poorly differentiated phenotype, MCT1-positive cancer cells are likely to harbor stem-like phenotypes in heterogeneous populations of tumor cells. After all, activated mitochondrial metabolism produces enough energy not only for self-renewal by proliferation but also for invasion/distant metastasis, both of which are activated in CSCs.

Thus, the pharmacological blockage of MCT1 is useful for the treatment of cancer. MCT1 inhibition disrupts metabolic symbiosis, and MCT1-positive aerobic cancer cells can no longer uptake lactate [20], which suggests that MCT1-positive CSCs play a fundamental role in maintaining the hierarchy in tumor cellular society, in contrast to MCT4-positive cells (Fig. 5).

### Acquisition of stem-like and malignant phenotypes with metabolic reprogramming

**Fig. 6:**
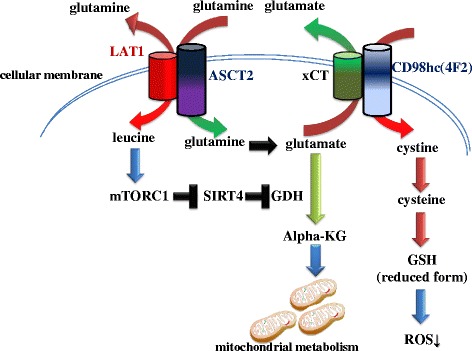
Metabolic reprogramming of amino acids due to coordinated transporters. ASCT2/LAT1 and xCT/CD98hc transporter complexes in tumor cells activate the mTORC1-SIRT4-GDH axis and glutathione synthesis, respectively. The former pathway promotes conversion of glutamate into alpha-KG, a substrate of the TCA cycle, whereas the latter pathway maintains redox status

The cooperation of amino acid transporters is necessary for cancer cells to undergo metabolic reprogramming and maintain stem-like phenotypes. For example, triple-negative breast cancer (TNBC) cells, which lack estrogen receptor, progesterone receptor, and the tyrosine kinase receptor HER2, exhibit addiction to glutamine metabolism due to coordination between the xCT and ASCT2 amino acid transporters [48, 49]: xCT uptakes cystine in exchange for glutamine, for use in GSH synthesis [7], whereas ASCT2 uptakes glutamine in a collaborative manner [50]. Glutamine is simultaneously imported via ASCT2 transporter and exported in exchange for leucine via the LAT1/4F2 (CD98 heavy chain) antiporter [48]. The glutamine uptake pathway contributes to the synthesis of alpha-KG, promoting the TCA cycle in mitochondria, as well as glutamate, thereby promoting synthesis of nucleotides required for cellular proliferation [48] (Fig. 6). Thus, metabolic reprogramming, which is orchestrated by the elevated expression and interaction of amino acid transporters, contributes to the activation of glutamine metabolic reprogramming and protects tumor cells against accumulation of oxidative stress mediated by cystine metabolic reprogramming.

Remarkably, circulating tumor cells (CTCs) that have undergone metabolic reprogramming provide themselves with a microenvironment that is favorable for colonization and distant metastasis. Recent work showed that CTCs derived from colon adenocarcinoma and positive for CD110, the thrombopoietin receptor, can home to the pre-metastatic niche and colonize metastatic hepatic tissue due to elevated lysine catabolism [51, 52]. Lysine degradation provides CD110-positive CTCs with glutamate and acetyl-CoA, which contributes to the synthesis of anti-oxidant GSH and p300-dependent LRP6 acetylation, respectively [52, 53]. This metabolic reprogramming promotes the metastatic potential of CTCs via a reduction in ROS levels, elevation of self-renewal potential, and activation of the Wnt/beta-catenin signal pathway [52]. Thus, CTCs resemble CSCs during the process of metastasis, at least in terms of the ‘education’ of the pre-metastatic niche. Most importantly, this metastatic phenotype is supported by lysine metabolic reprogramming.

A subpopulation of cancer cells that depend heavily on aerobic glycolysis robustly uptakes and consumes glucose, whereas another subpopulation engages in OXPHOS and glutaminolysis with activated mitochondrial metabolism. The efficiency of lactate production in the former (MCT4-positive) subpopulation is much higher than in the latter (MCT1-positive) subpopulation, which relies on OXPHOS and glutamine-derived TCA cycle in the mitochondria [54] (Fig. 5). Thus, tumor cells tend to decrease microenvironmental pH via elevated lactate secretion. The acidic tumor microenvironment induces expression of matrix metalloproteinases (MMPs), especially MMP-2 and MMP-9 [55]. Thus, metabolic reprogramming remarkably enhances the invasion and metastatic potentials of cancer cells.

### Activation of glutamine metabolism driven by oncogene addiction

Mitochondria plays a much more important role in cancer metabolism than previously expected, and glutaminolysis is the most common metabolic pathway regulated in this organelle [56]. Glutaminolysis is the series of biochemical reactions by which glutamine is catabolized into downstream metabolites, e.g., alpha-ketoglutarate (alpha-KG) and glutamate. Via the TCA cycle, alpha-KG undergoes catabolism to malate, which is transported into the cytoplasm and converted to pyruvate, and then ultimately to lactate [22]. Mechanistically, mTORC1 signaling promotes glutamine anaplerosis via upregulation of glutamate dehydrogenase (GDH) [57]. SIRT4 is a mitochondrial-localized member of the sirtuin family of NAD-dependent enzymes that play fundamental roles in metabolism, stress response and longevity [58]. In regard to glutaminolysis, SIRT4 is a critical negative regulator for glutamine metabolism in mitochondria [58], which is down-regulated at the transcriptional level when the mTOR signaling pathway is activated [57]. Thus, mTOR inhibitors such as rapamycin are expected to block mTORC1-SIRT4-GDH axis, which is essential for glutaminolysis [57] (Fig. 6).

As mentioned above, tumor tissue consists of a cellular population that is heterogeneous in terms of dependency on the Warburg effect and mitochondrial metabolism. Relative to slow-cycling CSCs, proliferative cancer cells tend to take up a great deal of glutamine, as well as glucose, for the generation of metabolites [54]. Both aerobic glycolysis and glutaminolysis are frequently simultaneously activated in malignant cancer cells [36, 59]. Seemingly paradoxically, however, some cancer cell lines cannot survive and proliferate in the absence of glutamine, despite the fact that glutamine is a non-essential amino acids that can be synthesized from glucose [60]. Glutamine is a primary substrate for the TCA cycle and is required to maintain the redox state via the production of nicotinamide adenine dinucleotide phosphate (NADPH). Glutaminolysis enables cancer cells to reduce NADP^+^ to NADPH, a reaction that is catalyzed by malic enzymes. NADPH is a required electron donor for reductive steps in lipid synthesis, nucleotide metabolism, and maintenance of reduced GSH [21]. In this way, metabolic reprogramming of glutaminolysis enables cancer cells to regulate redox state.

Oncogenic c-Myc mediates elevation of glutaminolysis in cancer cells. c-Myc promotes both glutamine uptake and glutamine catabolism [61]. Because of c-Myc-mediated metabolic reprogramming, cancer cells tend to exhibit “glutamine addiction” [48, 61]. This is a typical example of metabolic reprogramming in cancer cells with oncogene-addiction [62, 63], suggesting a potential “Achilles’ heel” of tumor cells that are addicted to glutamine metabolism in manner that is mediated by c-Myc.

### Therapeutic strategies targeting metabolic reprogramming

Drug repositioning (DR), screening for anti-cancer therapeutic effects of conventionally administered medications for non-malignant disorders, has attracted a great deal of attention because the safety and frequency of side effects of these medicines have been already proven [64]. Proton pump inhibitor (PPIs), for instance, are acid-activated pro-drugs that inhibit H/K-ATPase expressed in gastric parietal cells and are conventionally used for the treatment of gastric ulcer [65]. PPIs have exert synergistic effects on chemotherapy [66] by modulating the acidic microenvironment [67] or down-regulating microRNAs involved in chemotherapy resistance [68]. Other typical examples of DR include sulfasalazine [7, 8, 69], itraconazole [70, 71], terfenadine [72, 73], and simvastatin [74, 75] are described in Table 1. To address their anti-tumor therapeutic effects in clinical settings, all of those drugs are being tested in clinical trials or xenograft experiments.

**Table 1:** Typical examples of conventional drugs as anti-tumor agents

Name of agent	Conventional application	Mechanism of action	Clinical significance	Target
Sulfasalazine	Rheumatic arthritis, ulcerative colitis	Specific inhibition of xCT cystine transporter	Disruption of reduced glutathione (GSH) synthesis and to make cancer cells suscptible to oxidative stress	Gastric tumor and NSCLC progression, breast cancer metastasis
Itraconazole	Fungal infections such as Aspergillus	Inhibition of Smoothened (Smo), active Shh receptor	To inhibit proliferation of secondary mutated Shh signal (e.g. Gli2 amplification)	Difficult-to-cure medulloblastoma
Terfenadine	Auto-immune disorders such as allergic dermatitis	Histamine receptor H1 antagonist	To prevent secretion of VEGF from mast cells localized in hypoxic lesion, and to induce ROS-mediated apoptosis and autophagy of melanoma cells	Malignant melanoma
Simvastatin	Hyperlipidemia	Specific inhibition of HMG-CoA reductase	To prevent mutant p53 from activating mevalonate pathway for cholesterol synthesis	Breast tumor, ovarian cancer

Here, we will describe in detail the potential effects of metformin as an anti-cancer drug. DR has revealed, for example, that metformin, an oral drug widely used to treat type 2 diabetes mellitus (DM) [76], prevents tumor growth and development. A large number of retrospective clinical studies also show that metformin prevents carcinogenesis and improves clinical prognosis [77–79]. Metformin activates AMPK signal transduction, which not only decreases insulin resistance in type 2 DM [76] but also blocks AMPK-mediated mTOR activation even in CSCs [77]. mTOR signals are regulated by amino-acid transporters, characterized by the L-type amino acid transporter 1 (LAT1; SLC7A5) and the glutamine/amino acid transporter (ASCT2; SLC1A5) [80, 81], which is why the AMPK-mTOR axis functions as a sensor of dynamic change in the nutrient/growth factor microenvironment. In particular, leucine uptake via LAT1 activates the mTOR signal pathway [81, 82] leading to poor prognosis [83, 84]. Because EpCAM is a functional CSC marker that forms a complex with amino-acid transporters such as LAT1 [82, 85], it is reasonable that the LAT1 expression level would be positively correlated with poor prognosis [83, 84]. Therefore, the LKB1-AMPK-mTOR axis is orchestrated by amino-acid concentration in the tumor microenvironment, and this axis promotes metabolic reprogramming of cancer cells in response to the microenvironment.

Remarkably, recent investigations have revealed that this anti-type 2 DM drug suppresses ectonucleotide pyrophosphatase/phosphodiesterase family member 1 (ENPP1). Consequently, metformin can inhibit the generation of the subpopulation of cancer cells that express high levels of ABCG2, an ATP-binding cassette (ABC) transporter responsible for active drug efflux. Mechanistically, the cytosolic domain of ENPP1 is crucial for interaction with ABCG2 at the cellular membrane; thus ENPP1 contributes to drug resistance by promoting the stabilization of ABCG2 [86, 87]. In addition, metformin induces microRNA-27b-mediated suppression of ENPP1, which reduces chemoresistance and tumor seeding potential [86]. ENPP1 is widely accepted as a cause of insulin resistance in type 2 DM [88], emphasizing the significance of drug repositioning. Collectively, these observations indicate that this anti-DM agent is a promising means to attenuate the malignant behavior of cancer cells, much like other drugs conventionally administered for non-cancerous diseases.

## Conclusions

The complex and dynamic metabolic reprogramming should be regarded as a reflection of the “robustness” of tumor cells against unfavorable conditions. Hyper-adaptation due to metabolic reprogramming of cancer cells is likely to give us a great opportunity to attack the “shatter point” in heterogeneous tumor tissue. DR enables us to identify “silver bullets” for the treatment of tumor tissues in metabolically heterogeneous cell populations. To facilitate development of novel therapeutic strategies, the synergistic effects of repositioned drugs with conventional anti-cancer agents should be evaluated in clinical trials in the near future.

## Abbreviations

alpha-KG: Alpha-ketoglutarate
AMPK: Adenosine monophosphate-activated protein kinase
CAFs: Cancer-associated fibroblasts
CSC: Cancer stem-like cell
CTC: Circulating tumor cells
DM: Diabetes mellitus
DR: Drug-repositioning
ECM: Extracellular matrix
ENPP1: Ectonucleotide pyrophosphatase/phosphodiesterase family member 1
GDH: Glutamate dehydrogenase
HIF-1 alpha: Hypoxic inducible factor-1 alpha
LAT1: L-type amino acid transporter 1
MCT: Monocarboxylate transporter
MMP: Matrix metalloproteinases
MRD: Minimal residual disease
mTOR: Mammalian target of rapamycin
NADPH: Nicotinamide adenine dinucleotide phosphate
OXPHOS: Oxidative phosphorylation
ROS: Reactive oxygen species
TCA: Tricarboxylic acid
